# Findings of Cardiovascular Workup of Kidney Transplant Candidates: A Retrospective Study of a Single-Center in Saudi Arabia

**DOI:** 10.1155/2023/4653069

**Published:** 2023-10-10

**Authors:** Ziad Arabi, Mohammed H. Tawhari, Haneen S. Al Rajih, Talha M. Youssouf, Mohamad Y. Abdulgadir

**Affiliations:** ^1^Division of Nephrology, Department of Medicine, King Abdulaziz Medical City, Riyadh, Saudi Arabia; ^2^King Abdullah International Medical Research Center, College of Medicine, Riyadh, Saudi Arabia; ^3^King Saud Bin Abdulaziz University for Health Sciences, Riyadh, Saudi Arabia

## Abstract

**Background:**

There are limited data about the prevalence of cardiovascular (CV) risk factors and the findings of CV workup among kidney transplant (KTx) recipients (KTRs) in Saudi Arabia.

**Methods:**

A single-center retrospective study of KTRs who underwent KTx from 2017 to 2020 was performed. We reviewed the prevalence of CV risk factors and the results of the pre-KTx CV workup which was derived from the American Heart Association guidelines.

**Results:**

We included 254 KTRs. The mean age was 43.1 ± 15.9 years, and 55.5% were men and 79.5% were living-donor KTRs. Pre-emptive KTx was 9.8%, peritoneal dialysis was 11.8%, and hemodialysis was 78.3% (arteriovenous fistula: 33.1% versus hemodialysis catheter: 66.9%). The mean dialysis vintage was 4.8 ± 3.3 years for deceased-donor KTRs versus 2.4 ± 2.6 years for living-donor KTRs. CV risk factors were hypertension: 76%, diabetes: 40.6% (type 1 : 25.2% versus type 2 : 74.7%), hyperlipidemia (low-density lipoprotein >2.6 mmol/L): 40.2%, coronary artery disease (CAD): 12.6%, smoking: 9.1%, peripheral vascular disease: 2.8%, and cerebral vascular disease: 2.4%. The prevalence of obesity stage 1 was 19.7% and obesity stage 2 was 4%. Left ventricular hypertrophy was present in 38.5%. The ejection fraction was abnormal (<55%) in 22%. Abnormal wall motion was present in 34 patients (13.4%). A cardiac (PET-CT) stress test was conducted on 129 patients (50.8%) which showed abnormal perfusion in 37 patients (28.7%). Out of those who required PET-CT, 18.6% had a coronary artery calcium scoring (CACS) of more than 400, 41.8% had a CACS of zero, 29.4% had a CACS of 1–100, and 14.7% had a CACS of 100–400. Coronary angiogram was required in only 41 patients (16.1%), 12 (29.3%) required coronary interventions, 25 (61%) were treated medically, and 4 (9.8%) did not have any CAD. CT scans of pelvic arteries were performed in 118 patients (46.5%). It showed moderate or severe calcifications in only 7 patients (5.9%), whereas it was normal in 97 patients (82.2%), or it showed only mild calcifications in 14 patients (11.9%).

**Conclusion:**

This study outlines the prevalence of CV risk factors and the findings of the pretransplant CV workup among KTx candidates who underwent KTx. Multicenter national studies will be helpful to validate the generalizability of these findings.

## 1. Background

There are currently over 20,000 patients on dialysis in the Kingdom of Saudi Arabia (KSA) [1]. Additionally, there are 9,810 patients currently undergoing follow-up after kidney transplant (KTx) [1]. There were 4,471 new patients starting renal replacement therapy in 2020 with an average annual net increase of 6.2% [2, 3]. Of the total of 14,190 kidneys transplanted in KSA between 1979 and 2020, 76% were from living donors and 24% were from deceased donors [3].

Chronic kidney disease (CKD) is frequently associated with the traditional cardiovascular (CV) risk factors, such as diabetes mellitus (DM) and hypertension (HTN), and nontraditional CV risk factors such as anemia, hyperparathyroidism, uremia, hypervolemia, inflammation, and oxidative stress [4, 5]. By the time the patients require KTx, many of them have multiple CV risk factors [5].

Given the high prevalence and the atypical presentation of CV disease among KTx candidates [6, 7], multiple guidelines recommend CV screening to risk-stratify KTx candidates before surgery [8–12]. However, a large degree of variation exists among transplant centers, clinical practice patterns, and clinical guidelines regarding who should be screened and opinions are still based on mixed observational data with a large potential for bias [8–12]. The CV risk assessment of KTx candidates at our center, King Abdulaziz Medical Center, is based mostly on the American Heart Association (AHA) guidelines 2013 [10, 13], which is also in line with the most recent KDIGO Guideline 2020 [11, 12].

Additionally, data on the CV risk factors and findings of the CV screening among KTx candidates in Saudi Arabia and the Middle East are limited. Hence, in this study, we examined the prevalence of CV risk factors and the findings of CV workup of KTx candidates at our center to estimate the CV burden among kidney transplant recipients (KTRs) in KSA.

## 2. Methods

A single-center retrospective study of KTx candidates who underwent KTx from January 2017 to May 2020 at King Abdullah International Medical Research Center, Riyadh, KSA, was conducted. After obtaining approval from the Institutional Review Board (IRB) (NRC21R/390/09), we retrospectively reviewed the prevalence of CV risk factors and the results of the pretransplant CV workup.

At our center, screening echocardiograms are performed on all KTx candidates. A cardiac stress test (PET-CT) is performed on those who have ≥3 CV risk factors, limited functional status, or abnormal echocardiogram findings. The decision to perform a coronary angiogram differs from cardiology's assessment. In addition, abdominal/pelvic CT scans with intravenous contrast are performed to evaluate the extent of pelvic arterial calcifications (AC) and atherosclerosis in most KTx candidates. Patients on PD and those who are considered for preemptive transplantation or at a low surgical risk are typically excluded from abdominal/pelvis CT scanning [13].

All patients who successfully underwent KTx at our center from 2017 to 2020 were included in our analysis. Patients who declined KTx or were transferred to another center before KTx were excluded from the study. The collected data include the demographics of the KTRs and their traditional CV risk factors (HTN, DM, BMI, CAD, CVA, PVD, and smoking), nontraditional risk factors (parathyroid hormone (PTH) and hemoglobin), and the results of KTx workup including echocardiogram, PET-CT scan, abdominal/pelvic CT scan, and coronary angiogram results (if applicable).

## 3. Statistical Analysis

In this current study, we used SPSS version 26 to analyze our data. Descriptive statistics were reported as mean and standard deviation for normally distributed continuous variables and median and interquartile range for nonnormally distributed continuous variables. Categorical variables were reported as frequencies and percentages. To compare continuous variables between two groups, we used the Mann–Whitney *U* test for nonnormally distributed variables. All *p* values were two-tailed, and a *p* value of less than 0.05 was considered statistically significant.

## 4. Results

### 4.1. The Characteristics and CV Risk Factors of the KTRs

A total of 254 KTRs were included. The mean age was 43.1 ± 15.9 years, and 55.5% were men and 79.5% were living donor KTRs. Preemptive transplantation was 9.8%, PD was 11.8%, and hemodialysis (HD) was 78.3% (arterial venous fistula: 33.1% vs. hemodialysis catheter (hemodialysis catheter): 66.9%).

HTN was the most common CV risk factor among our cohort (76.4%), and over half of them (52.4%) had uncontrolled HTN defined by BP >140/90. DM was the second most common CV risk factor (40.7%) with 75% of the patients with DM having type 2 DM. Other CV risk factors among KTx candidates were hyperlipidemia defined by LDL >2.6 mmol/L or 100 mg/dL (40.2%), CAD (12.6%), smoking (9.1%), PVD (2.8%), and CVA (2.4%). The prevalence of obesity stage 2 (BMI 35–39.9 kg/m^2^) was 4% and obesity stage 1 (BMI: 30–34.9 kg/m^2^) was 19.7%.  Table 1 outlines the characteristics and CV risk factors of the KTRs.

The mean dialysis vintage was 4.8 ± 3.3 years for deceased-donor KTx versus 2.4 ± 2.6 years for living-donor KTx. Dialysis vintage also varied based on the blood group (Table 2).

Anemia which was defined as hemoglobin <130 g/L in men and 120 g/L in women was present in 67.7% with a mean Hgb of 114.3 ± 17.3. The mean PTH was 67.8 ± 54.7 (normal: 12.75 pmol/L).

### 4.2. The Findings of CV Workup of Kidney Transplant Recipients

Left ventricular hypertrophy (LVH) (mostly mild) was the most common abnormality in our pre-KTx workup (38.5%), followed by abnormal EF defined as EF <55% (22%), and further followed by abnormal wall motion abnormalities (mostly global dyskinesia) which was present in 13.4%.

Cardiac stress testing (PET-CT) was performed on 129 patients (50.8%), and it showed abnormal perfusion in 28.7% of those who had stress tests. Out of those who underwent PET-CT, 24 patients (18.6%) had a calcium score of more than 400, 54 patients (40%) had a calcium score of zero, 38 patients (29.4%) had a calcium score of 1–100, and 19 patients (14.7%) had a calcium score of 100–400.

A coronary angiogram was required for only 41 (16%) patients. Among those who required coronary angiograms, 12 patients (29.3%) required interventions, 25 patients (61%) received medical therapy, and 4 patients (9.8%) had normal coronaries.

CT scans of the abdomen and pelvis were performed on 118 patients (46.5%) to assess the suitability of the pelvic vasculatures for transplant. Out of the 118 patients who underwent CT scans, only 7 patients (5.9%) had severely calcified vessels, while the remaining scans showed either mild calcifications (11.8%) or normal studies (82.2%).

Table 3 shows the findings of the CV workup of KTRs.

**Figure 1:**
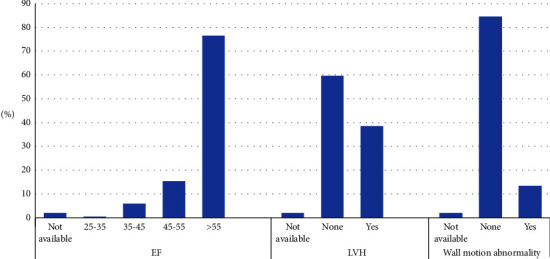
Finding of screening pre-KTx echocardiography.

**Figure 2:**
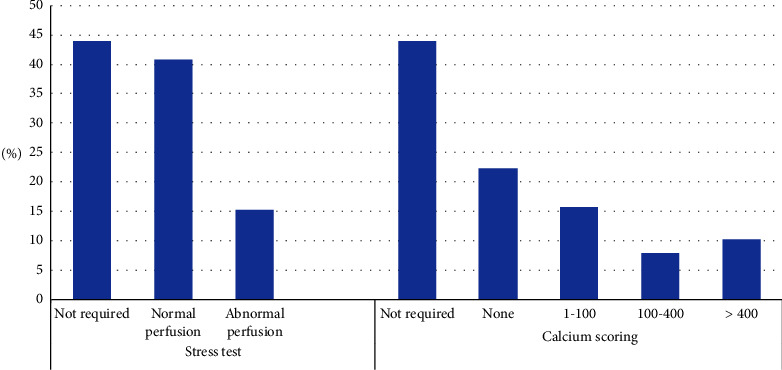
Finding of screening pre-KTx cardiac PET-CT stress test (cardiac perfusion and CACS).

**Figure 3:**
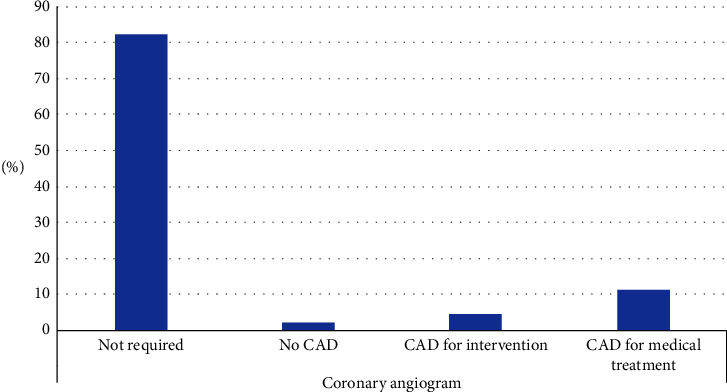
Finding of a pre-KTx coronary angiogram.

**Figure 4:**
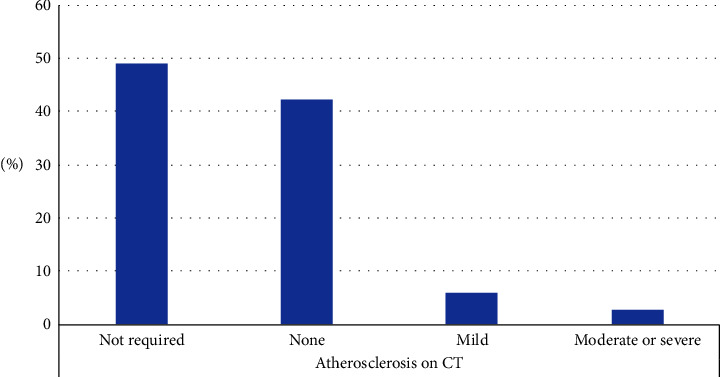
Finding of pre-KTx screening CT angiogram of pelvic arteries.

Figures 1–4 show the findings of pre-KTx screening echocardiography, cardiac PET-CT stress test (cardiac perfusion and CACS), coronary angiogram, and CT angiogram of pelvic arteries, respectively.

## 5. Discussion

CV disease is the most common cause of death and hospitalization among end-stage kidney disease (ESKD) patients in KSA and worldwide [14–17]. In KSA, HD patients have high overall rates of hospitalization (11.6 days/patient/year) and mortality (8.07 deaths per 100 patients per year) [18]. CV causes (51.7%) unknown/sudden death (27.5%) and fluid overload are the leading causes of death in these patients [18]. KTx decreases the CV risk among these patients [14, 15]; however, the risk remains elevated compared to the general population. In fact, CV risk is particularly high in the early post-KTx period, and up to 50% of death within the first 30 days post-KTx is related to CV events [14, 16–20]. Multiple traditional and nontraditional risk factors contribute to this increased risk after KTx [21]. The prevalence of CV risk factors varies based on patients' characteristics, etiology of primary kidney disease, type of transplantation, and dialysis vintage. Similarly, the findings of CV workups vary based on the screening protocol used by each center. In spite of a relatively high number of KTx performed in KSA since 1979 [3], there are only scant published data about the CV risk assessment of the KTx candidates.

This study reviews the CV risk factors of KTRs and the results of their CV workup at a major tertiary transplant center in KSA. Before discussing the CV risk factors and the findings of pre-KTx workup, there are a few points that are worth noting. First, this study demonstrates that our KTRs are relatively young. This finding is consistent with previous reports that showed that patients in KSA develop ESKD at a relatively younger age than those in Western countries (30% of ESKD patients in the KSA are less than 40 versus 15% in the USA) [3, 13]. Typically, ESKD patients remain dialysis-dependent unless they undergo KTx [13]. However, KTRs are usually younger than the general HD population in KSA (43 versus 51–53 years) [18, 22]. This is likely due to the community's efforts to have younger patients transplanted as soon as possible.

Second, the high rate of living-donor KTx at our center is consistent with the rate (76%) reported nationally [1, 3]. Yet, the rate of preemptive KTx in KSA is about four times higher than the rate in the USA (9.8 versus 2.5% only) [23]. Also, our results showed that ABO blood groups have a major impact on waiting time on deceased donors. We believe that improving the living-donor KTx, particularly for those with certain blood groups, can help shorten the dialysis vintage and subsequently lower the CV risk among KTRs. Note that, the waiting times indicated in our cohort represent the waiting time for those who were successfully transplanted. However, the overall waiting time for the patients on the waiting list is much longer. Third, consistent with previous reports, the most common causes of ESKD among KTx candidates at our center are DM (25%), unknown (23%), and glomerulonephritis (21%) [13]. These factors are likely to be the leading causes of ESKD of KTRs in KSA.

Our current study demonstrates the traditional and nontraditional CV risk factors among the Saudi KTRs. The most common traditional risk factors among our cohort are DM, obesity, HTN, dyslipidemia, smoking, and established CV diseases (CAD, CVA, and PVD). The nontraditional risk factors include anemia and hyperparathyroidism.

DM, one of the most common traditional CV risk factors, is very prevalent in KSA. Previous studies from KSA noted that DM starts at a younger age leading to higher rates of diabetic microvascular and macrovascular complications [24, 25]. In addition, type 1 DM seems to be overrepresented among KTx candidates (10% vs 4.2% of the general HD population), and our study supports this finding [26]. Improving diabetes control and implementing screening and management of diabetic kidney disease can potentially lower the rate of CKD progression and subsequently may improve the CV disease burden.

Our study revealed that the rate of obesity among our KTx candidates is comparable to the rate reported in the general population in KSA [27]. However, previous reports have shown that the rate of obesity among Saudi HD patients is lower (11%–23%) [26, 28]. This is probably due to malnutrition among many dialysis patients, particularly those with longer dialysis vintage, whereas KTx candidates constitute a selected healthier subgroup with a better nutritional status. It is also important to note that many KTx centers place their KTx candidates with a high BMI on hold till their weight is optimized to a BMI of ≤35 or ≤37 [29].

Consistent with previous reports, our study demonstrates that HTN is very prevalent among KTx candidates and the control rate remains suboptimal [28]. A better HTN control, by adjusting the volume status and titrating the antihypertensive medications, can potentially modify the CV risk. In addition, dyslipidemia is also common among our KTRs and the control rate is similar to those reported among ESRD in KSA (50%) [28].

The rate of smoking among our KTRs is 9%, which is comparable to the previous reports from KSA [18, 26, 30]. However, this rate is less than the reported rate of smoking among the general Saudi population, where smoking is reported at an overall rate of 19.8% (30.0% in men and 4.2% in women) [31]. However, the rate of smoking among our KTRs is comparable to the rate of smoking among American KTRs (11%) [32, 33]. Note that, smoking cessation is highly encouraged but smoking is not considered an absolute contraindication of KTx at our center. Measures to promote smoking cessation among the CKD population can potentially lower the CV risk.

CAD and CVA are common among HD patients in KSA with a prevalence as high as 11–32.7% and 3.3–11.8%, respectively [18, 26, 28]. The prevalence of PVD among patients with ESKD in KSA is about 6.4% [28]. These rates are much less among the selected KTRs. For example, in our cohort of KTRs, PVD was present in only 2% of our participants. This is likely because KTx candidates with advanced vascular diseases are often declined.

Anemia and hyperparathyroidism are the most common nontraditional risk factors among our KTRs. According to a national, multicenter study of 389 HD patients throughout KSA, anemia and hyperparathyroidism were controlled in only 50% and 23%, respectively [28]. Selected KTx candidates may have more optimized control of their anemia and hyperparathyroidism. Our results support these findings.

Hemodialysis catheter use among HD patients in KSA is high (31%) [22]; however, the rate of hemodialysis catheter use among KTx candidates is even much higher. This is likely due to the tendency to defer arteriovenous fistula (AVF) creation in KTx candidates with available living-donor KTx. Hemodialysis catheters may be considered as a viable bridge therapy option in patients with living donor availability [34]. However, pre-KTx dialysis duration may be quite long even when planned with a living donor [35]. The advantages of protecting these patients from AVF creation must be carefully evaluated against catheter-related risks [35].

As mentioned earlier, the results of the CV workup of KTR candidates vary among centers, based on the studied population and the screening protocol. The CV risk assessment of KTx candidates at our center is derived mostly from AHA guidelines 2013 [13, 36].

Our study is the first study in KSA to report the pre-KTx echocardiographic findings even among those who were successfully transplanted. The most common echocardiographic findings in our cohort are LVH (38.5%), reduced EF (22%), and wall motion abnormalities (13.4%).

LVH is an adaptive response to increased cardiac work, typically caused by combined pressure and volume overload, resulting in cardiomyocyte hypertrophy and increased intercellular matrix and it is as common as 75% of incident dialysis patients [37]. ESKD patients with heart failure (HF) and reduced EF have higher morbidity and mortality rates when compared with those without HF [37].

We found only two studies addressing echocardiographic findings among ESKD patients in KSA and no other previous studies among KTRs were found. Albeshri et al. studied 333 Saudi HD patients and demonstrated that LV dysfunction (EF <40) was associated with higher morbidity and mortality rates when compared with patients with normal EF [38]. In another single-center study of 192 Saudi patients, Sharabas et al. showed that PD patients had higher urine output and better Kt/V values, however, they were more edematous and using more antihypertensive medications and had a lower EF [30]. In a study of 356 Spanish patients with CKD awaiting KTx, the most common echocardiographic findings were LVH in 68.5%, decreased EF in 7.6% (EF 55%–45% in 3.1%, EF 45%–30% in 2.8%, EF <30% in 1.7%), and myocardial contractility alterations in 8.1% [39].

It should be noted that previous reports have shown that LVH was reported in almost 70% of transplant candidates, which is almost twice as high as our study [39, 40]. This could be due to the lower average age of our KTRs (43.1 versus 54.3 years) [40]. Also, the availability of living donors that allowed for a higher rate of preemptive transplantation could have contributed to a lower LVH rate by shortening the HD vintage and subsequently lowering the uremia-induced cardiac dysfunction.

Among KTx candidates, LVH, decreased EF, and resting wall motion abnormalities may be related to uremia or CAD [8]. At our center, all KTx candidates with a lower EF require cardiologist assessment before KTx. If EF improves to 35% or more, most patients can proceed to transplantation. If EF remains between 25%–35%, we intensify the dialysis regimen for 1 month [41–43] and we proceed with KTx if EF improves. If there is no improvement, we obtain cardiology and anesthesia consultations and proceed if the patient accepts high-risk surgery. Patients with EF <25% remain on hold till EF improves [13]. Note that, many of these echocardiographic findings (LVH and EF) improve quickly, within a month following KTx. However, the diastolic dysfunction may not improve significantly [40].

Cardiac PET-CT is considered the standard screening tool for KTx candidates with risk factors as mentioned above [13]. However, different transplant centers in KSA utilize different CV screening protocols and imaging modalities. At our center, we follow AHA criteria, and a cardiac PET-CT stress test is performed on those who have at least 3 CV risk factors, limited functional status, or abnormal echocardiogram findings [13]. The current study represents the first study in KSA to report cardiac PET-CT results of KTx candidates according to the inclusion criteria of the AHA guidelines. Our results showed that a stress test was required in about 50% of KTx candidates who were successfully transplanted and there were abnormal perfusion results in about a third of those who required it.

We found only one study in KSA by Fathala et al. where asymptomatic ESKD patients referred for KTx at a major tertiary center in KSA were evaluated by a single-photon computed tomography (SPECT) imaging. The incidence of myocardial perfusion defects was only 13% [44]. However, patients with established CAD (having prior percutaneous coronary intervention, coronary bypass surgery, or prior myocardial infarction) were excluded. Note that, PET-CT imaging has a much higher sensitivity over SPECT for the diagnosis of CAD [45, 46]. However, multiple international studies have reported a prevalence of myocardial perfusion abnormalities in ESRD ranging from 27% to 45% based on their population and the inclusion criteria of their local protocol [47–49].

In this study, CACS was performed along with cardiac PET-CT in 125 patients (53.1%). Out of those who underwent the CACS test, 54 patients (43.2%) had a calcium score of zero and 24 patients (18.6%) had a calcium score of more than 400. The absence of CACS in the general population is associated with a low prevalence of obstructive and nonobstructive CAD; and a CACS of zero can serve as a “gatekeeper” for more advanced imaging in the general population [50]. On the other hand, CACS is common and more severe in patients with ESKD and can also occur in the absence of occlusive coronary atherosclerosis [51]. In ESKD, calcium deposits are not only found in advanced atherosclerotic plaques, as typically seen in the general population, but they also can be deposited heavily in the arterial media [52]. CAC also increases arterial stiffness and subsequently increases the CV risk [51, 53, 54].

In ESKD, CACS >0 is seen in 83% of patients, and it has a specificity of only 53% for predicting obstructive CAD [55, 56]. Using a higher threshold (CACS >400), may increase its specificity to 77% but may lower the sensitivity to 67% [57]. A cut-off score of CACS has not been wildly validated among patients with ESKD [51, 56, 58, 59]. Nevertheless, CACS can provide additional information for CV risk stratification in KTRs [59, 60].

To the authors' knowledge, this current study is the first study in KSA to report CACS among KTx candidates who were successfully transplanted. In addition, our study is also the first study to report the findings of coronary angiogram among KTx candidates in KSA when applying the AHA protocol.

We found only two studies that reported the findings of coronary angiograms among KTx candidates in the Gulf area. The first study was from a major tertiary center in KSA by Mohamed et al. who retrospectively studied the prevalence of CAD in an asymptomatic cohort of KTx candidates on HD. All included patients underwent coronary angiograms without noninvasive testing. They included 368 patients with a mean age of 56 years; of which 25.3% had a prior diagnosis of CAD, 6.8% had previous coronary artery bypass grafting (CABG), 8.4% had previous coronary stents, 78% had DM, 9.5% had prior KTx, 95.7% were on dialysis for >1 year, and 2.5% had a stress test positive for ischemia [61]. Out of the 368 patients, 45% had CAD, 17% had 3-vessel disease, 11% had 2-vessel disease, 5.2% had significant left main artery narrowing, and 17% had single-vessel disease. The patients with significant 3-vessel disease or left main artery involvement underwent revascularization; and 19% underwent coronary artery bypass grafting, 5% had stenting of the coronary arteries, and 4.7% were on maximal medical therapy [61]. This study showed that significant stenosis of coronary arteries is very prevalent among patients with ESKD who were referred for KTx [61].

The second study was from Qatar by Ali et al. who prospectively studied 75 ESKD patients, who subsequently underwent KTx. Their prespecified protocol utilized noninvasive and/or invasive tests for the evaluation. The median age of their cohort was 51 years. Overall, 21 (28%) patients showed evidence of CAD, an incidence that was much higher among patients with DM (81%). There were 13 (17%) subjects shown to have CAD by coronary angiogram in the absence of a background CAD history [62].

Our results indicate that by applying AHA guidelines among our KTRs, a coronary angiogram was required only in a limited number of patients (16%) and coronary intervention was required only in less than a third of these cases. The lower rate of CAD among our KTRs is likely due to the younger age of our study participants and the fact that our study included KTx candidates who were healthy enough to get transplanted. To clarify, patients with ESKD who are not referred or excluded from KTx, potentially due to a high CV risk, were not included in the analysis, which can underestimate the overall rate of CAD among the ESKD population. Also, the higher rate of preemptive KTx among our cohort may have lowered the dialysis vintage, meaning that more patients transplanted before they accumulated CV risk. It should be noted that the overall prevalence of CAD in ESKD patients in KSA and internationally can reach up to 20–34% [30, 63, 64].

CKD patients have an increased incidence of arterial calcifications (AC). Risk factors include age, male gender, HD, hyperparathyroidism, DM, and dyslipidemia [65, 66]. The severity of pelvic AC may impact the surgical planning of KTx, choice of anastomosis site, complexity of the surgery, and eventually patients and grafts survival [65, 67]. AC is recognized as a good biomarker of the overall CV burden [65].

Žuža et al. retrospectively studied 118 KTRs who underwent pretransplant pelvic CT. Calcification morphology, circumference, and length of both common and external iliac arteries were scored. The pelvic calcium score was calculated as the total score sum of all three calcification features in all vessels. AC in at least one vascular segment was found in 79% of patients. The extent of iliac artery calcification in KTx recipients quantified by pelvic calcium scoring on pretransplant CT correlates with graft and overall patient survival [66].

We found only one retrospective study about AC among 836 HD patients in KSA. The mean duration since the first diagnosis of CKD was 8.1 ± 6.3 years and mean dialysis vintage was 5 ± 4.6 years. The mean age of patients was 51.8 ± 15.4 years. The prevalence of AC was 40.8% [26]. However, CT of pelvic arteries was not utilized in this study as it is of more prognostic relevance to KTx. In our study, the lower rate of calcification in pelvic vessels supports the possibility that our cohort was healthier than the average ESKD population in KSA. Again, the younger age and the relatively shorter dialysis vintage because of living donor availability, may have led to such a lower rate.

An international, randomized control study to evaluate the prognostic value of iliac AC determined with CT in patients with KTx is currently underway [68]. There is also a need to utilize a standardized pelvic calcification score based on the assessment of the common iliac artery and external iliac artery calcifications, based on their morphology, circumference, and length as proposed by previous studies [68].

In summary, KTRs have a significant burden of CV risk factors. The prevalence of the findings of their CV workup may vary among centers according to the protocol used.

CV risk assessment of KTx candidates helps to identify patients with CV risk factors that are amenable to interventions that may lower the potential postoperative complications. Screening may also help to exclude very high-risk patients with a nonmodifiable disease which may lead to premature graft and patient loss [19, 69].

In this study, we reviewed the prevalence of CV risk factors and the findings of the CV workup of the KTx candidates at our center as an estimate of the CV disease burden among KTRs in KSA. This study has several strengths and limitations. First, its single-center, retrospective nature may limit its generalizability. In addition, it included only patients who successfully underwent KTx and did not include those who were not referred for KTx or were declined after workup, which may have underestimated the burden of CV diseases among KTx candidates.

Nevertheless, this study is the first to highlight in detail the description of CV risk assessment and the findings of CV workup among KTRs in KSA. Many of these areas have not been previously reported. It is also the first study in KSA to apply and report the findings according to the AHA guidelines and consensus guidelines of cardiovascular risk assessment in kidney transplantation in KSA [13, 36]. This step is very essential to create a unified national approach for the CV risk assessment of KTx candidates and to enable a future multicenter national study to verify these findings and determine their prognostic values.

## 6. Conclusion

Although KTRs are a selected subgroup of the ESKD population, they still carry a significant burden of CV risk factors. This study outlines the prevalence of CV risk factors among renal transplant candidates along with the findings of the pretransplant CV workup in a single transplant center in KSA. Having a unified national approach to the CV risk assessment of KTx candidates among KTx centers in KSA is necessary to enable a multicenter national study which can verify these findings and determine their prognostic values.

## Figures and Tables

**Table 1:** Characteristics and CV risk factors of KTRs.

	Total 254
Age (mean ± SD years)	43.1 ± 15.9
Age ≥30 years	190 (74.8%)
Age <30 years	64 (25.2%)
*Gender*	
Female	113 (44.5%)
Male	141 (55.5%)
*Type of dialysis*	
Hemodialysis	199 (78.3%)
Peritoneal dialysis	30 (11.8%)
Preemptive KTx	25 (9.8%)
*Type of hemodialysis access*	
Arteriovenous fistula	84 (33.1%)
Permcath	170 (66.9%)
*Type of transplant*	
Deceased donor	52 (20.5%)
Living donor	202 (79.5%)
Hypertension	194 (76.4%)
Diabetes (DM)	103 (40.6%)
DM type 1	26 (25.2)
DM type 2	77 (74.7%)
Hyperlipidemia (LDL >2.6 mmol/L)	102 (40.2%)
Smoking	23 (9.1%)
Coronary artery disease	32 (12.6%)
Peripheral vascular disease	7 (2.8%)
Cerebral vascular disease	6 (2.4%)
Dialysis vintage (mean ± SD years)	3.1 ± 3
Deceased donor KTx	4.8 ± 3.3
Living donor KTx	2.4 ± 2.6
Dialysis vintage >2 years	104 (40.9%)
Body mass index (BMI)	25.4 ± 5.7
Underweight	27 (10.7%)
Normal weight	93 (36.6%)
Overweight	73 (28.7%)
Obesity stage 1	50 (19.7%)
Obesity stage 2	10 (4%)
Systolic blood pressure (SBP)	138.7 ± 21.8
Diastolic blood pressure (DBP)	77.8 ± 14.9
Uncontrolled blood pressure (BP >140/90)	133 (52.4%)
Parathyroid hormone (PTH) (NL: 12.75 pmol/L)	67.8 ± 54.7
Hemoglobin (Hgb) (g/L)	114.3 ± 17.3
Anemia (Hgb <130 g/L in men and 120 g/L in women)	172 (67.7%)

**Table 2:** Dialysis vintage and ABO blood groups.

	Total	Median (interquartile range) (years)	Deceased	Median (interquartile range) (years)	Living	Median (interquartile range) (years)	*P* value
O	120 (47.2%)	2 (1 to 4)	17	4 (2.5 to 6.5)	103	2 (1 to 3)	0.002
A	75 (29.5%)	1 (1 to 3)	15	3 (1 to 5)	60	1 (1 to 2)	0.011
B	63 (24.8%)	3 (1 to 7)	16	7 (3 to 10)	47	2 (1 to 4)	0.002
AB	17 (6.6%)	2 (1 to 4.5)	7	4 (1 to 5)	10	1 (1 to 2.25)	0.070

**Table 3:** Findings of CV workup of KTRs.

		Total 254
*Echocardiography*		
*Ejection fraction (EF)*		
>55		195 (76.8%)
45–55		39 (15.4%)
35–45		15 (5.9%)
25–35		1 (0.4%)
NA		4 (1.6%)
*Ejection fraction (EF)*		
<55		55 (22%)
≥55		195 (78%)
*Left vertical hypertrophy (LVH)*		
None		152 (59.8%)
Yes		98 (38.5%)
Not available		4 (1.6%)
* Wall motion abnormalities*		
None		216 (85%)
Yes		34 (13.4%)
Not available		4 (1.6%)
*Coronary calcium scoring*		
Not required	119 (46.9%)	
Zero	54 (21.3%)	
1–100	38 (15%)	
100–400	19 (7.5%)	
More than 400	24 (9.4%)	
*Cardiac nuclear perfusion stress test*		
Not required	119 (46.9%)	
NL perfusion	98 (38.5%)	
Abnormal perfusion	37 (14.6%)	
*Coronary angiogram*		
Not required	213 (83.9%)	
No CAD	4 (1.6%)	
CAD present, for coronary intervention	12 (4.7%)	
CAD present, for medical treatment only	25 (9.8%)	
*Atherosclerosis on CT of pelvic arteries*		
Not required	136 (53.5%)	
None	97 (38.2%)	
Mild	14 (5.5%)	
Moderate/severe	7 (2.8%)	

## Data Availability

The data used to support the findings of this study are included within the article.

## Abbreviations

AC: Arterial calcifications
AHA: American Heart Association
AVF: Arteriovenous fistula
BMI: Body mass index
CAD: Coronary artery disease
CAC: Coronary artery calcification
CACS: Coronary artery calcium scoring
CKD: Chronic kidney disease
CV: Cardiovascular
CVA: Cerebral vascular disease
DM: Diabetes mellitus
EF: Ejection fraction
ESKD: End-stage kidney disease
HD: Hemodialysis
HTN: Hypertension
KSA: Kingdom of Saudi Arabia
KTx: Kidney transplantation
KTRs: Kidney transplant recipients
LDL: Low-density lipoprotein
LVH: Left ventricular hypertrophy
PD: Peritoneal dialysis
PET-CT: Positron emission tomography and computed tomography scans
PTH: Parathyroid hormone
PVD: Peripheral vascular disease
SPECT: Single-photon emission computed tomography.
